# Supplementary material for: Factors associated with intrauterine contraceptive device use among women of reproductive age group in Addis Ababa, Ethiopia: A case control study
Source: PLoS One. 2020 Feb 18;15(2):e0229071. doi: 10.1371/journal.pone.0229071 (PMC7028271; doi:10.1371/journal.pone.0229071)
Supplement: S1 File — (PDF) [file pone.0229071.s001.pdf]

MCM MYUNGSUNG CHRISTIAN MEDICAL CENTER

MMC MYUNGSUNG MEDICAL COLLEGE

Research and Ethics Committee

Date: March 21, 2017

Ref: MMC/EC/563/2017

To: Intern Dr. Biruk Engida

RE: Research Ethics Committee Decision

Dear Intern Dr. Biruk,

We would like to inform you that the MCM/MMC Research Ethics Committee (Institutional Review Board) on its meeting held on March 15, 2017 has reviewed your study entitled "*Assessment of factors affecting use of Intra-uterine contraceptive device (IUCD) among women of reproductive age group seeking family planning services at health centers in Addis Ababa, Ethiopia 2016*".

This proposal has been approved by our committee, thus allowing you to start working on the subsequent steps of your project.

We expect a full report on the findings of your study once it is finished, and wish you good success in this important endeavour.

Sincerely,

Roger P. Holland MD, PhD

Chair, MMC / MCM Research Ethics Committee

Scanned by CamScanner
