# Supplementary material for: Factors associated with intrauterine contraceptive device use among women of reproductive age group in Addis Ababa, Ethiopia: A case control study
Source: PLoS One. 2020 Feb 18;15(2):e0229071. doi: 10.1371/journal.pone.0229071 (PMC7028271; doi:10.1371/journal.pone.0229071)
Supplement: S1 Appendix — (DOCX) [file pone.0229071.s002.docx]

## Information Sheet

Good Morning/Afternoon, my name is ________________________. I am a trained data collector for the research **“Factors affecting use of Intra-uterine contraceptive device (IUCD) among women of reproductive age group seeking family planning services at Addis Ababa”**. This survey is to identify the factors affecting the use of IUCD among women of reproductive age group between 15 and 49. The research will be helpful to tackle the factors that affect the use of IUCD and will help us to develop strategies in improving the family planning services.

The interview will take about 25 minutes. Your name and other personal identifiers will not be recorded on data collection form and the information that you give us will be kept confidential. The information you provide will not be used for another purpose than this study. If you don’t want to answer some or all of the questions, you have the right to do so. If you fill discomfort with the interview, please fill free to drop it any time you want. However, your willingness to answer all questions would be appreciated.

Could I have your permission to continue? A. YES B. NO

If yes, continue with the interview.

If no, thank the woman and skip to the next FP client.

**Interviewer who collect the consent**

Name_______________________ Signature ___________________

## Questionnaire

**Code _____________**

**Health center______________________ Date of Interview________________**

**Part 1. Socio-demographics characteristics**

1. Age of the respondent (in years)

1.15- 20 years 2. 21- 30 years 3. 31-40 years 4. 41-49 years

1. What is your current marital status?

1. Unmarried 2. Married 3. Divorced 4. Widowed

1. What is your religion?

1. Orthodox 2. Muslim 3. Catholic 4. Protestant

5. Other (Specify) _________________

1. What is your Educational level?
2. Can’t write and read
3. Can write and read
4. Primary school (1-8th grade)
5. Secondary school (9th -12th grade)
6. Higher education
7. Don’t know

1. What is your Husband’s/ Partner Educational level?
2. Can’t write and read
3. Can write and read
4. Primary school (1-8th grade)
5. Secondary school (9th -12th grade)
6. Higher education
7. Don’t know
8. What is your current occupation? 1. House wife

2. Unemployed

3. Student

4. Government employee

5. Private employee/NGO

6. Own business

7. Daily laborer

8. Other (specify)____________

1. How much is your monthly income? 1. less than 500 birr

2. 500-999 birr

3. 1000-1499birr

4. 1500-1999birr

5. more than 2000birr

6. No income

**Part II. Reproductive characteristics**

1. Have you ever been pregnant? 1. Yes 2. No
2. If yes in Q 9, how many times you got pregnant? _______ times
3. If yes in Q9, were all your pregnancies planned? 1. Yes 2. No
4. Have you ever encountered abortion? 1. Yes 2. No
5. If yes in Q 11, how many abortions you had encountered? ______
6. If yes in Q 11, what type/s of abortion you had experienced? 1. Induced 2. Spontaneous
7. Have you ever given birth? 1. Yes 2. No
8. How many children ever born to you? Total____ Male____ Female____
9. How old is your youngest child? _____ months/ years old
10. Do you wish to have children in the future? 1. Yes 2. No
11. If yes in Q 19, how many years do you want to wait before having the next pregnancy/child?

1. 2-3 years 2. 4-5 years 3. 5-10 years

**Part III. Contraceptive Knowledge, Attitude and Practice**

1. Which method of modern contraception do you know? (more than one answer can apply)

1. Pills 2. Implant 3. Injectable 4. IUCD 5. Condom

6. Diaphragm 7.Female sterilization 8.Male sterilization

9. Other, specify………..

1. What is your source of Information about family planning methods? (more than one answer can apply)

1. Health facilities 2. TV 3. Radio

4. Magazines 5. Internet 6. Friends

7. nowhere/no one 8. Other (specify) _________________

1. Have you ever heard of IUCD? 1. Yes 2. No
2. Have you ever seen IUCD? 1. Yes 2. No
3. Do you know how IUCD is inserted into the uterus? 1. Yes 2. No
4. Do you know where you can obtain IUCD?

1. Hospital (health center) 2. Health extension worker

3. Private clinic 4. Pharmacy

5. Supermarket 6. Don’t know

7. it’s impossible to obtain 8. Other (specify) _______________

1. Are you comfortable by exposing your private organ during insertion of IUCD?

1. Yes 2. No

1. From what you know about IUCD, do you think you would recommend it to a friend or relative in case of need? 1. Yes 2. No
2. Which type of contraceptive method are you currently using?

1. OCP 2. Injectable 3. IUCD

1. Why do you choose the contraceptive you are currently using?

1. No need of daily motivation 2.Husband approval 3.No menstrual bleeding

4. No weight gain 5.Highly effective in preventing pregnancy 6.Told by a friend to use 7.No alternative method I know

8. Chosen by the service provider 9. Other, specify ……………………………….

1. . For how long have you used this contraceptive? (For OC& Injectable users only)

…… months or………years

1. What do you feel about the contraceptive method you are currently using?

1. Effective in preventing pregnancy 2.Easy to use 3.Reversible

4. No side effects 5. Other, specify………………………….

**PART IV: PERCIEVED MYTHS AND MISCONCEPTION**

1. Do you think that IUCD causes infertility?

1. Yes 2.I don’t think 3. I don’t know

1. Do you think that IUCD can cause infection?

1. Yes 2.I don’t think 3. I don’t know

1. Do you think that IUCD can cause cancer?

1. Yes 2.I don’t think 3. I don’t know

1. Do you think that IUCD use affects sexual intercourse?

1. Yes 2.I don’t think 3. I don’t know

1. Do you think that IUCD can result in pregnancy while in the uterus?

1. Yes 2.I don’t think 3. I don’t know

1. Do you think that IUCD may migrate to other organs beyond the uterus?

1. Yes 2.I don’t think 3. I don’t know

**PART V: COMMUNITY AND METHOD RELATED FACTORS**

1. What is your partner/husband opinion towards IUCD use?
2. Supporting 2. Against 3. Do not know 4. Not accessible
3. What is your friends/peer opinion towards IUCD use?

1. Supporting 2. Against 3. Do not know

1. What are the possible reasons you are not using IUCD? (For OC& Injectable users only)

1. Can result in pregnancy while it is in the uterus

2. It migrates to other parts of the body

3. Irregular bleeding during menstruation

4. Headaches

5. Increased risk of Pelvic Inflammatory Disease

6. Perforation occurs in the uterus

7. Pain in Pelvic region 8. Others _______________________________

**PART VI: SERVICE RELATED FACTORS**

1. Have you been told about IUCD as an option of FP method in this health facility?

1. Yes 2.No

1. Have you been told about the availability of IUCD in this health facility?
2. yes 2.No
3. Have you been told about the availability of health professional capable of inserting IUCD in this health facility?

1. Yes 2.No

1. Have you been told in this health facility that IUCD use need minimal follow up after insertion?

1. Yes 2.No

1. Do you have access to IUCD whenever you need it? 1. Yes 2.No

**THANK YOU FOR PARTICIPATION!**
