# Supplementary material for: Factors associated with intrauterine contraceptive device use among women of reproductive age group in Addis Ababa, Ethiopia: A case control study
Source: PLoS One. 2020 Feb 18;15(2):e0229071. doi: 10.1371/journal.pone.0229071 (PMC7028271; doi:10.1371/journal.pone.0229071)
Supplement: Attachment — Submitted filename: Reviwers Comment MM PONE-D-19-22523.docx [file pone.0229071.s006.docx]

 Reviewer: Mengistu Meskele

Date: 05 October 2019

Title: **Factors Associated with Intrauterine Contraceptive Device Use among Women of Reproductive Age Group in Addis Ababa, Ethiopia: A Case Control Study**

Manuscript number: PONE-D-19-22523

**General comments**

Generally, this research title currently a public health important in Ethiopia. Though, it needs further improvement in order to adhere the publication criteria of the journal. My comments are presented below

The study presents the results of original research= Yes
2. Results reported have not been published elsewhere=Yes
3. Experiments, statistics, and other analyses are performed to a high technical standard and are described in sufficient detail. No
4. Conclusions are presented in an appropriate fashion and are supported by the data. No
5. The article is presented in an intelligible fashion and is written in Standard English. No, some improvement is needed.
6. The research meets all applicable standards for the ethics of experimentation and research integrity. Not separately explained in the method section, not ethics approval letter attached
7. The article adheres to appropriate reporting guidelines and community standards for data availability =Yes

**Specific comments**

- Title and sub-titles should be sentence case and follow journal style of formatting manuscript
- The formatting styles of tables in this manuscript are not suitable for publication. Each variable should be written in cell or should follow the journal style. Also one table should not be presented two to three pages. If possible, it would be each could be table to present in one page?
- Table 4. COR and AOR should be presented along with 95% CI in one cell?
- In table 1,2 and 3 the COR and 95% CI columns should be removed?
- Abstract: should not be more than 300 words?
- Why the definition of cases was only selected from new users of family planning? On other hand your controls were selected both from new and return clients? Definition and selection criteria for cases and controls should be the same. What is your justification for this difference?

From line 123, From all the health centers in Addis Ababa, 20 health centers (two from each sub-city) were selected by employing simple random sampling technique?

- Question: How many health centers were there in AA? Why only 20 were selected?
- Unnecessary capitalization and inconsistency in use of abbreviations are identified. For instance, intrauterine Device/IUCD. Sometimes you use IUCD but other time intrauterine Device. If once you abbreviated it in the first time use, use IUCD throughout?
- Did you check the model fitness test? That means post estimation test? How did you controlled over matching? What about multicolloniarity?
- Letter for Ethical clearance file should be attached as supporting information?
- List of abbreviations should be removed from line numbers 324-331?
- Discussion: You didn’t consider negative finding in your discussion. You discussed only positive finding. If possible consider it.
- Strengths and limitations should be stated at end of discussions?
- Presentation of 95% CI: You put it not consistently in your document throughout, sometimes you used one digit, and other times 2 digits. Be consistent and I recommend 2 digits. It is usually preferable and be consistent?
- *Line 236: You used p-*value <0.20 in the bivariate analysis. Why p- value < 0.2? Why not 0.25?. If you could have used backward conditional stepwise regression rather than enter method, your multivariable analysis will be very sound, also your table also cannot three to four pages. Consider this?
- References are not completed and an acceptable label.
- References 2, 3,8,9,10,11,16,17,18,22,23,28,29,30,31, are either not completed or URL and accessed dates are missed, also the authors naming are not correct. List of more than 6 authors, you should write the first 6 authors and use et al.?
- Financial disclosure, conflict of interest and source of funding are not mentioned.

**Conclusion**

- In the conclusion section, the statement “For the fertility rates continue to decline, unintended pregnancies, unsafe abortions and 317 associated mortalities to be reduced the use of contraceptives plays an important role”. This sentence can be removed and the author can bring conclusions from his/her finding rather than stating the above unrelated issues of the study.
