# Supplementary material for: Factors associated with intrauterine contraceptive device use among women of reproductive age group in Addis Ababa, Ethiopia: A case control study
Source: PLoS One. 2020 Feb 18;15(2):e0229071. doi: 10.1371/journal.pone.0229071 (PMC7028271; doi:10.1371/journal.pone.0229071)
Supplement: Attachment — Submitted filename: Response to reviewers.docx [file pone.0229071.s007.docx]

**Point by point response**

PONE-D-19-22523
Factors Associated with Intrauterine Contraceptive Device Use among Women of Reproductive Age Group in Addis Ababa, Ethiopia: A Case Control Study

Dear editor and reviewers,

Thank you for your comments and suggested revisions that have helped us to improve the manuscript. We have revised our manuscript according to your constructive and very insightful comments and suggestions. Attached you will find our revised manuscript and below you will find a response to each of your comments.

**Response for editor’s comment**

In addition to points raised by the reviewers, I would suggest that the authors re-structure the Discussion to discuss topics in order based on strength of association. Partner support was the strongest determinant of selecting an IUCD but is only discussed midway through this section. The limitations section also do not encompass the full range of limitations, included reported nature of many of the co-variates, the non-representative sample selection and use of non-matched controls that further limit generalizability; please make this section more robust. The manuscript would benefit from review by a native English speaker for editing and flow and the Methods section can be made more concise.

**Response: Thank you for the suggestion. The discussion is re-structured now to discuss the strongest predictor first. Also, limitations of the study is now detailed. The revised manuscript is also reviewed by native English speaker.**

**Response for reviewer 2 comments and suggestions**

R2: Title and sub-titles should be sentence case and follow journal style of formatting manuscript

**Response: Thank you, now it is corrected.**

R2: The formatting styles of tables in this manuscript are not suitable for publication. Each variable should be written in cell or should follow the journal style. Also one table should not be presented two to three pages. If possible, it would be each could be table to present in one page?

**Response: Thank you for the comment. The formatting of the tables are now corrected.**

R2: Table 4. COR and AOR should be presented along with 95% CI in one cell?

**Response: Thank you, COR and AOR are now presented along with 95% CI in one cell.**

R2: In table 1,2 and 3 the COR and 95% CI columns should be removed?

**Response: Thank you for the suggestion. COR and 95% CI are removed from the tables.**

R2: Abstract: should not be more than 300 words?

**Response: Thank you, it is corrected.**

R2: Why the definition of cases was only selected from new users of family planning? On other hand your controls were selected both from new and return clients? Definition and selection criteria for cases and controls should be the same. What is your justification for this difference?
From line 123, From all the health centers in Addis Ababa, 20 health centers (two from each sub-city) were selected by employing simple random sampling technique?

**Response: Thank you for the comment. Actually, we have included only new cases and controls to reduce the potential biases. Now, this is corrected throughout the revised manuscript.**

**Twenty health centers out of the total 98 health centers were selected by simple random sampling technique (lottery method).**

R2: Question: How many health centers were there in AA? Why only 20 were selected?

**Response: There were 98 health centers in Addis Ababa. To increase the scope and ensure representativeness of the population attending care, we randomly selected 20 health centers (two from each sub-city). It was not feasible to take more than 20 health centers. We employed simple random sampling technique to ensure equal chance of being included in the study.**

R2: Unnecessary capitalization and inconsistency in use of abbreviations are identified. For instance, intrauterine Device/IUCD. Sometimes you use IUCD but other time intrauterine Device. If once you abbreviated it in the first time use, use IUCD throughout?

**Response: Thank you. IUCD is used throughout the manuscript.**

R2: Did you check the model fitness test? That means post estimation test? How did you controlled over matching? What about multicolloniarity?

**Response: Yes, we have checked model fitness test by Hosmer-Lemeshow goodness-of-fit test. Also, we have checked multicoliniarity by using the multicoliniarity diagnostics (VIF and Tolerance test).**

R2: Letter for Ethical clearance file should be attached as supporting information?

**Response: Attached as supporting information**

R2: List of abbreviations should be removed from line numbers 324-331?

**Response: Thank you, addressed.**

R2: Discussion: You didn’t consider negative finding in your discussion. You discussed only positive finding. If possible consider it.

**Response: Thank you. We have now considered discussing pertinent negative findings in the discussion.**

R2: Strengths and limitations should be stated at end of discussions?

**Response: We have detailed the strengths and limitations of the study**

R2: Presentation of 95% CI: You put it not consistently in your document throughout, sometimes you used one digit, and other times 2 digits. Be consistent and I recommend 2 digits. It is usually preferable and be consistent?

**Response: Thank you for the comment. We have now put the numbers consistently**

R2: Line 236: You used p-value <0.20 in the bivariate analysis. Why p- value < 0.2? Why not 0.25?. If you could have used backward conditional stepwise regression rather than enter method, your multivariable analysis will be very sound, also your table also cannot three to four pages. Consider this?

**Response: Thank you for the comment. We used P value <0.25 as a criteria to enter variables into the multi-variable model. Also, we have now re-analyzed our data using the backward conditional stepwise binary logistic regression model and the findings are reported.**

R2:  References are not completed and an acceptable label.
⎫ References 2, 3,8,9,10,11,16,17,18,22,23,28,29,30,31, are either not completed or URL and accessed dates are missed, also the authors naming are not correct. List of more than 6 authors, you should write the first 6 authors and use et al.?

**Response: Thank you, the formatting of the references is now corrected.**

R2: Financial disclosure, conflict of interest and source of funding are not mentioned.

**Response: Based on the PLOS ONE submission guidelines, statements on financial disclosure and conflict of interest were given in the submission form in the system.**

R2: In the conclusion section, the statement “For the fertility rates continue to decline, unintended pregnancies, unsafe abortions and 317 associated mortalities to be reduced the use of contraceptives plays an important role”. This sentence can be removed and the author can bring conclusions from his/her finding rather than stating the above unrelated issues of the study.

**Response: The introductory sentence of the conclusion is used to link our findings with its public health importance. Then, we have concluded the study based on the findings of the study.**
