# Supplementary material for: Factors associated with intrauterine contraceptive device use among women of reproductive age group in Addis Ababa, Ethiopia: A case control study
Source: PLoS One. 2020 Feb 18;15(2):e0229071. doi: 10.1371/journal.pone.0229071 (PMC7028271; doi:10.1371/journal.pone.0229071)
Supplement: Attachment — Submitted filename: Reviewer MM comment 17 December 2019.docx [file pone.0229071.s008.docx]

**Reviewer: Mengistu Meskele**

**Date: 17 December 2019**

**Title: Factors Associated with Intrauterine Contraceptive Device Use among Women of Reproductive Age Group in Addis Ababa, Ethiopia: A Case Control Study**

Manuscript number: PONE-D-19-22523

General comments

Generally, in this time the author made good improvement and the paper look sound.However , I do have very few comments. Still the author didn’t address the journal style of font size for headings. Table 5. Cross tabulation for cases and controls are missed. Kindly include these issues?

1. The study presents the results of original research= Yes

2. Results reported have not been published elsewhere=Yes

3. Experiments, statistics, and other analyses are performed to a high technical standard and are described in sufficient detail. Yes

4. Conclusions are presented in an appropriate fashion and are supported by the data. Yes

5. The article is presented in an intelligible fashion and is written in Standard English. Yes

6. The research meets all applicable standards for the ethics of experimentation and research integrity. Yes

7. The article adheres to appropriate reporting guidelines and community standards for data availability =Yes

Minor comments

1. Contribution: Equal or unequal contribution should be explained at first page?
2. Style: Font style for title and headings should be adhered journal style?
3. Table 5. Cross tabulation for cases and controls should be included?
