# Supplementary material for: Factors associated with intrauterine contraceptive device use among women of reproductive age group in Addis Ababa, Ethiopia: A case control study
Source: PLoS One. 2020 Feb 18;15(2):e0229071. doi: 10.1371/journal.pone.0229071 (PMC7028271; doi:10.1371/journal.pone.0229071)
Supplement: Attachment — Submitted filename: Response to reviewers.docx [file pone.0229071.s009.docx]

**Point by point response**

PONE-D-19-22523
Factors Associated with Intrauterine Contraceptive Device Use among Women of Reproductive Age Group in Addis Ababa, Ethiopia: A Case Control Study

Dear editor and reviewers,

Thank you for your comments and suggested revisions that have helped us to improve the manuscript. We have revised our manuscript according to your constructive and very insightful comments and suggestions. Attached you will find our revised manuscript and below you will find a response to each of your comments.

**Response for editor’s comment**

**Editor:** The authors have been largely responsive to previous comments but both reviewers and I note that the authors disregarded responding to several comments in the first review. There was no response to Reviewer 1's critiques; Reviewer 2 noted several comments that did not receive a response, and I note that the Limitations section still does not include some major limitations that were brought to the authors' attention in the first review, namely the reported nature of several of the covariates (e.g., literacy) and the absence of matched controls. Further, though you state the revised manuscript was reviewed by a native English speaker, there remain multiple places where verb tense, punctuation, and other grammatical errors are present or examples of awkward phrasing (which can be adjusted and reduce word count in the abstract). For example: "In each health center, all the eligible cases were included in the study consecutively until the desired sample size was achieved. For each case, two adjacent controls were selected to be included in the study." can be improved to "In each selected health center, all eligible cases were enrolled consecutively until sample size was achieved. Two consecutive controls were selected for each case." Last, there appear to be different fonts within the manuscript and portions do not comply with formatting requirements. Please attend to all of these matters completely and in the response letter, please state what was done in response and provide illustrative text rather than stating the query was addressed, as this was not consistently the case with the last revision. Thus, you are invited to revise and re-submit.

**Response: Thank you for the comments. The prior request sent to us was to review comments/suggestions based on the comments from the editor and reviewer 2. That is why we didn’t reply for the reviewer 1 questions. Now, we have replied to the reviewer 1 queries too.**

**The limitations of the study is also further detailed to address the** **reported nature of the covariates and the absence of matched controls. The study might be also limited by social-desirability bias with regard to reporting the myths and miss-conceptions towards IUCD and the literacy level of the participants. Absence of matched controls (e.g., in terms of age) might have also affected the findings of this study.**

**Consistency of font styles and formats are addressed now. To further improve the overall flow of the manuscript, it is reviewed by a native English speaker.**

**Response for Reviewer 1 comments**

R1: 1- Abstract: No need to state the sample sizes twice in the abstract.

**Response: Thank you, it is addressed. Sample size is mentioned once in the revised manuscript.**

R1:  Modify the results and/or conclusion to indicate that the significant factors in the multivariate logistic model were INDEPENDENT predictors of IUCD use.

**Response: Thank you, this is indicated now in the revised manuscript. The adjusted odds ratio also indicates that the predictors are independently associated with the IUCD use after the confounding factors are controlled.**

R1: Line 121: Provide additional details for justifying the study’s target sample size. The power calculation should include: sample size, alpha level (indicating one or two-sided), minimal detectable difference and statistical testing method.

**Response: Thank you for the comment. It is addressed and all the entities for sample size calculations were given.**

R1: Tables: Define all abbreviations and provide only the overall p-values rather than pairwise p-values for variables with more than two categories.

**Response: Thank you for the suggestion. All abbreviations defined. The overall p-values were given in the tables.**

R1: Tables: Include percentages corresponding to the frequencies.

**Response: Thank you, percentages are now included.**

R1: When discussing the results of the multivariate model, clarify that four factors were significant when adjusted for the nonsignificant factors that were also included in the model. This has been stated properly in the discussion, but should also be included in the results section of the abstract.

**Response: Thank you for the comment. Statement clarifying that the associated factors were significant when adjusted for the nonsignificant factors that were also included in the model.**

**Response for Reviewer 2 comments**

R2: Contribution: Equal or unequal contribution should be explained at first page?

**Response: Thank you. It is included now. All the authors contributed equally to this work.**

R2: Style: Font style for title and headings should be adhered journal style?

**Response: Thank you. Font styles are adhering to the journal style.**

R2:  Table 5. Cross tabulation for cases and controls should be included?

**Response: Thank you, it is included now.**
